# Does supervision matter? A randomized controlled study of laparoscopic simulator training in undergraduate education

**DOI:** 10.3389/fsurg.2026.1815062

**Published:** 2026-05-11

**Authors:** Ioannis Gkekas, Magnus Edblom, Emil Forsberg, Anders Ringnér, Asif Halimi, Urban Arnelo, Gabriel Sandblom, Lars Enochsson

**Affiliations:** 1 Department of Diagnostics and Intervention, Surgery, Umeå University, Umeå, Sweden; 2 Department of Nursing, Umeå University, Umeå, Sweden; 3 Department of Health Sciences, Karlstad University, Karlstad, Sweden; 4 Division of Surgery, Department of Clinical Science, Intervention, and Technology (CLINTEC), Karolinska Institutet, Stockholm, Sweden; 5 Department of Clinical Science and Education, South General Hospital, Karolinska Institutet, Stockholm, Sweden; 6 Division of Orthopaedics and Biotechnology, Department of Clinical Science, Intervention, and Technology (CLINTEC), Karolinska Institutet, Stockholm, Sweden

**Keywords:** laparoscocpy, medical student, RCT - randomized controlled trial, simulator training, supervision, teacher education

## Abstract

**Background:**

Simulation-based training is a cornerstone of surgical education, yet evidence regarding the comparative impact of continuous instructor supervision vs. independent practice for novices remains limited. This study aimed to assess the efficacy of continuous, remote video-guided supervision compared with independent training in laparoscopic simulation for medical students.

**Methods:**

A randomized controlled trial was conducted with 20 medical students having no prior laparoscopic experience. Participants were randomized to either a supervision group (*n* = 8), receiving real-time video-guided instruction, or an independent group (*n* = 12) engaging in self-directed practice. Training was performed using the Simball Box simulator (“Rope Race” task). Outcome measures included objective task completion times and subjective ratings of perceived difficulty and feedback value via questionnaires.

**Results:**

Both groups demonstrated significant improvement in procedure times from warm-up to the first exercise (Supervised: *p* < 0.001; Independent: *p* = 0.020). While no statistically significant difference was found between groups in the primary outcome (performance time in the first unsupervised Rope Race test) the supervised group trended towards faster completion in early exercises. Notably, the supervised group reported significantly lower perceived difficulty post-training (*p* = 0.049), whereas the independent group perceived a significant reduction in the value of simulator-only feedback (*p* = 0.003).

**Conclusion:**

Remote real-time supervision significantly reduces perceived difficulty and prevents the devaluation of feedback compared to independent practice. These findings support a hybrid curricular model where resource-intensive supervision is front-loaded to facilitate early psychomotor skill acquisition, followed by independent volume training.

## 1 Introduction

Simulation-based training has established itself as a cornerstone of surgical education in the early stages of training, providing a safe, controlled environment in which trainees can acquire technical skills before performing procedures on patients. Laparoscopic surgery demands advanced psychomotor aptitude and visuospatial awareness – skills that often prove challenging for novices to master. Consequently, virtual reality (VR) and box trainers have been widely adopted to bridge the gap between theoretical knowledge and operative competence.

Numerous studies have demonstrated that structured simulator training enhances performance and mitigates error rates in laparoscopic tasks. Notably, Ahlberg et al. (1) demonstrated that proficiency-based VR training significantly reduced error rates during initial laparoscopic cholecystectomies performed by surgical residents. Furthermore, Enochsson et al. (2) highlighted the predictive role of visuospatial ability and gaming experience in simulator performance, suggesting that individual factors significantly influence learning curves.

Among undergraduate medical students, research indicates that simulator training enhances technical skills even in the early stages of surgical education. Ahlborg et al. (3), demonstrated that individualized feedback to medical students during laparoscopic simulator training resulted in shorter instrument path lengths and improved task efficiency. Similarly, tele-mentoring interventions have been shown to augment precision and performance when compared with self-directed practice (4). These findings underscore the critical importance of guidance and feedback within simulation-based learning.

In another randomized clinical trial, Seymour et al. (5) reported that VR-trained residents exhibited significantly superior performance in laparoscopic cholecystectomy compared to those lacking systematic simulator training. In a randomised clinical trial involving sixteen surgical trainees, Grantcharov et al. (6) demonstrated that those who underwent VR simulator training significantly improved their surgical performance in the operating theatre compared to the control group.

Recent advances in simulation-based education have increasingly focused on structured, adaptable, and evidence-based training frameworks. A randomized trial by Pan et al. (7) demonstrated that a laparoscopic training course designed around an evidence-based pedagogical model significantly improved operative performance among surgical trainees, underscoring the value of structured curricula in modern skills acquisition. Similarly, Zhou et al. (8) reported that a highly simulated and adaptable training system in a high-volume teaching hospital improved laparoscopic skill development among residents, highlighting the growing integration of flexible, technology-enhanced training environments. These recent findings complement the evolving landscape of remote and hybrid simulation approaches, reinforcing the relevance of evaluating supervised vs. independent training models in undergraduate learners.

Despite these advances, evidence remains limited regarding the comparative impact of continuous instructor supervision of undergraduate students vs. independent practice. Determining whether real-time guidance provides measurable benefits over self-directed training is crucial for optimising resource allocation and designing effective simulation programmes.

The objective of this randomised controlled trial was to assess the efficacy of continuous, video-guided, instructor-supervised training at a distance vs. independent training in laparoscopic simulation for medical students. We hypothesised that real-time guidance would yield superior technical proficiency, as confirmed by shorter task completion times and greater self-confidence among medical students.

## 2 Material and methods

### 2.1 Study design

This prospective, randomised controlled trial was conducted at Umeå University, Sweden, to evaluate the effect of continuous remote video-guided instructor supervision vs. no supervision during laparoscopic simulator training among medical students.

### 2.2 Participants

Initially, forty medical students were planned for inclusion based on a power analysis. However, due to slow recruitment, the study was terminated early after enrolling 20 students. Inclusion criteria were enrolment in the medical programme and no prior laparoscopic surgery experience. Exclusion criteria included previous simulator training or operative exposure. Written informed consent was obtained from all participants.

### 2.3 Sample size and power calculation

In a previous study (unpublished data), we found that students who underwent the test with guidance improved rope test performance by 2.1 mm/s, compared with a control group that improved by 0.6 mm/s (4). The standard deviation of the first group was 1.8 mm/s. Assuming an equivalent difference, a total of 36 trainees would be required to achieve 70% power to detect a statistically significant difference between the groups. A *post-hoc* power analysis was performed based on the observed effect sizes in procedure time comparisons between groups. Using the actual sample size of 20 participants, the statistical power achieved ranged from approximately 28% to 35%, depending on the comparison. This confirms that the study was underpowered relative to the *a priori* estimate of 70% power requiring 36 participants.

### 2.4 Pre-training questionnaire

The pre- and post-training questionnaires were adapted from previously published instruments used in laparoscopic simulation research and were reviewed by two senior surgeons and one educational specialist to ensure face and content validity.

Before randomisation, participants completed a structured questionnaire assessing:

- **Demographics:** Age, sex, and handedness.
- **Previous experience:** Prior use of endoscopic/laparoscopic simulators and the number of sessions.
- **Gaming habits:** Hours per week and type of games played.
- **Attitudes and expectations:** Likert-scale items regarding perceived difficulty, usefulness of simulator training, receptiveness to feedback, and ability to follow verbal instructions.

### 2.5 Randomisation

Participants were randomized in a 1:1 allocation ratio using a computer-generated blocked randomization sequence. Block sizes of 4, 6, and 8 were used and varied at random to prevent prediction of assignment. Allocation concealment was ensured through an automated randomization procedure generated and stored by an independent researcher not involved in participant enrollment or assessment. Group assignments were disclosed only after participants completed the baseline questionnaire to maintain concealment.

#### 2.5.1 Simulator and tasks

Training was performed on the Simball™ Box simulator (Surgical Science Sweden AB, Gothenburg, Sweden). Each participant completed the following:

- **Warm-up and training:** All participants, irrespective of group allocation, commenced with a warm-up exercise comprising the “Rope Race” procedure. This task involves using laparoscopic instruments within the Simball™ Box to pass a thread through eight loops arranged in a circle. The total time required to complete the exercise was recorded. Subsequently, participants practiced the procedure for 20 min, with or without remote video-guided supervision.
- **Final test tasks:** All participants, irrespective of group allocation, performed three final Rope Race procedures without supervision. The times for these three exercises were recorded.

#### 2.5.2 Post-training questionnaire

Immediately following the final tasks, participants completed a post-training questionnaire to evaluate perceived difficulty, the utility of the training, the value of the feedback received, and attitudes towards future simulator use.

### 2.6 Outcome measures

#### 2.6.1 Objective outcomes

- Task completion time (seconds)

#### 2.6.2 Attendee perceived outcomes

- Pre- and post-training questionnaire scores regarding perceived difficulty, feedback value, and motivation.

The primary outcome was the completion time of the first of the three unsupervised Rope Race tests (Test 1), performed immediately after the training intervention. This time point was chosen *a priori* to capture the immediate effect of supervision on early performance. Completion times for Tests 2 and 3 were predefined as secondary outcomes reflecting subsequent performance trajectory. For key outcomes, 95% confidence intervals (CIs) were calculated to provide estimates of the precision of between-group differences.

### 2.7 Data collection

The simulation exercises performed at Umeå University were recorded and subsequently assessed by an independent observer (LE) regarding completion time. The recording equipment setup followed the protocol described in a previous study by the group (4). Questionnaires were administered prior to and following the training session. Performance metrics were automatically logged by the simulator software but were, unfortunately, not saved. A temporary malfunction in the simulator's local storage buffer prevented the automatic export of kinematic metrics (e.g., instrument path length, angular movement, error counts). These data could not be recovered. However, task completion time was available through video recording review and remained usable as the primary performance measure.

#### 2.7.1 Statistical analysis

Continuous variables were expressed as mean ± SD or median (IQR) and compared using independent t-tests or Mann–Whitney U tests. Categorical variables were analyzed using chi-square tests. Correlations between baseline variables (e.g., gaming habits, attitudes) and performance were explored using Spearman's rank correlation. A *p*-value < 0.05 was considered statistically significant.

#### 2.7.2 Ethical considerations

The study was approved by the Swedish Ethical Review Authority (Ref. no. 2023-02208-01). Written informed consent was obtained from all participants.

## 3 Results

### 3.1 Participant flow and characteristics

Twenty medical students were randomized into two groups: continuous instructor supervision (*n* = 8) and independent practice (*n* = 12). Approximately 70% of participants were female, reflecting current medical school demographics. At baseline, there was no significant difference in task completion time between the supervision and independent groups (Table 1).

**Table 1:** Baseline characteristics.

Covariable	Supervised (*n* = 8)	Independent (*n* = 12)	Total (*n* = 20)	*P* value
**Demographics**				
**Sex**				
Male	2 (25.0%)	4 (33.3%)	6 (30.0%)	0.690
Female	6 (75.0%)	8 (66.7%)	14 (70.0%)	
**Playing computer games**				
No	4 (50.0%)	8 (66.0%)	12 (60.0%)	0.582
Yes	4 (50.0%)	4 (33.0%)	8 (40.0%)	
**Type of game -Shooter**				
No	5 (62.5%)	10 (83.3%)	15 (75.0%)	0.292
Yes	3 (37.5%)	2 (16.7%)	5 (25.0%)	
**Mobile**				
No	5 (62.5%)	8 (66.7%)	13 (65.0%)	0.292
Yes	3 (37.5%)	4 (33.3%)	7 (35.0%)	
**Age (years)**				
Mean (SD)	24.4 (2.7)	25.6 (4.9)	25.1 (4.1)	0.487
**Baseline task times**				
Seconds [Mean (SD)]	388 (109)	347 (172)	363 (148)	0.529

### 3.2 Primary outcomes: final sharp exercises

The primary outcome (completion time in Test 1) did not differ significantly between groups. Performance was assessed across the three final unsupervised tests. Test 1 served as the primary outcome, while Tests 2 and 3 were secondary outcomes.

Matched-pair analysis showed that both groups, irrespective of assignment, demonstrated a significant improvement in terms of shorter procedure times between the warm-up trials and the first exercise: the Supervised group (388s vs 168s; *p* < 0.001) and the independent group (347s vs 213s; *p* = 0.020). Comparisons between the groups at each recorded time point revealed no statistically significant differences. However, procedure times for both the first and second exercises tended to be slightly shorter in the supervised group compared with the independent group (*p* = 0.108 and *p* = 0.124, respectively). In the third exercise, this trend was no longer evident (Table 2). The supervised group completed Test 1 in a mean of 168 s (SD 47), compared with 213 s (SD 72) in the independent group. The mean difference was −45 s, with a 95% CI ranging from −108 to +18 s, indicating that the estimate was imprecise and did not reach statistical significance (*p* = 0.108).

**Table 2:** Within-group and between-group analyses (clean version).

Outcome	Supervised (Mean ± SD)	Independent (Mean ± SD)	Within-group *Δ* (s)	Within-group p	Between-group p
Warm-up	388 (109)	347 (172)	-	-	0.529
Test 1	168 (47)	213 (72)	Supervised: −220		
Independent: −134	Supervised: *p* < 0.001				
Independent: *p* = 0.020	0.108				
Test 2	156 (68)	217 (102)	-	-	0.124
Test 3	154 (58)	210 (141)	-	-	0.240

Within-group values reflect paired Warm-up → Test 1 analyses. Between-group values represent independent comparisons (Supervised vs Independent).

### 3.3 Secondary outcomes

#### 3.3.1 Perceived difficulty and training experience

Participants in the group randomised to supervised training reported significantly lower perceived difficulty after training compared to pre-training (4.1 vs 3.5; *p* = 0.049), whereas this was not the case in the independent group (Table 3).

**Table 3:** Pre/post questionnaire results.

	Supervised				Independent			
Questions in the Pre/Post Questionnaire	Before TRAINING	After	difference	*P* value	Before	After	difference	*P* VALUE
	(mean)	(mean)	(mean)		(mean)	(mean)	(mean)	
I think the simulator training **will be/was** difficult.	4.1	3.5	−0.6	0.049	3.6	3.7	0.1	0.838
I feel that the simulator training **will make/made** my final simulator test easier.	4.8	4.9	0.1	0.351	4.3	4.5	0.2	0.550
I think my experience with video games **will improve/improved** my performance.	4.0	3.1	−0.9	0.155	3.2	2.8	−0.4	0.175
I consider myself to be receptive to feedback-I found value in the feedback from either the video instruction or the simulator.	4.5	4.6	0.1	0.684	4.6	2.6	−2.0	0.003

Values shown as means. Differences are (After−Before) within group; *p*-values from paired tests per group.

Furthermore, participants in the independent group did not perceive value in the feedback provided solely by the simulator, as the post-training score was significantly reduced to half of the baseline value (4.6 vs 2.6; *p* = 0.003) (Table 3). However, pre- and post-training scores for the corresponding question in the supervised group did not change significantly, remaining at the same level (4.5 vs 4.6; *p* = 0.685).

##### 3.3.1.1 Subgroup analyses

###### 3.3.1.1.1 Gaming experience

Participants reporting computer gaming experience completed examination test 3 in a mean of 137 s, compared to 249 s for those without such experience (*p* = 0.063) (Table 4). Those who reported playing shooter games completed test 3 significantly faster than those who did not (130 ± 13 s vs 207 ± 130 s; *p* = 0.037). No statistically significant difference in completion time was observed in the tests between participants with experience playing mobile games and those with no such experience, although there was a trend toward faster completion in test 3 (*p* = 0.102) (Table 4).

**Table 4:** Rope race completion times relative to gaming experience.

Rope Race completion times (second)					
Phase	Mean	SD	Mean	SD	*P* value
	**Gaming experience *n* = 11**			**No gaming experience *n* = 9**	
Warm-up	333	127	401	171	*p* = 0.341
Live test 1	197	62	192	75	*p* = 0.856
Live test 2	168	70	223	112	*p* = 0.225
Live test 3	137	22	249	155	*p* = 0.063
	**Playing shooter games *n*** **=** **5**			**Do not play shooter games *n*** **=** **15**	
Warm-up	344	135	370	156	*p* = 0.728
Live test 1	205	83	191	63	*p* = 0.755
Live test 2	153	82	206	95	*p* = 0.269
Live test 3	130	13	207	103	*p* = 0.037
	**Playing mobile games *n*** **=** **7**			**Do not play mobile games *n*** **=** **13**	
Warm-up	312	124	391	157	0.238
Live test 1	191	42	197	78	0.814
Live test 2	173	61	203	107	0.425
Live test 3	142	24	212	139	*0.101*

### 3.4 Sex differences

Regarding Rope Race completion times, we observed no statistically significant difference between the sexes; however, there was a trend for men to complete live test 3 slightly faster (140 ± 33 s vs 208 ± 134 s; *p* = 0.092) (Table 5). No significant differences were found in Warm-up (*p* = 0.627), Test 1 (*p* = 0.639), or Test 2 (*p* = 0.233). Upon pairwise analysis of Rope Race times between the warm-up procedure and test 1, we found that women improved their times significantly (173 s; *p* = 0.001) whereas men showed a less pronounced improvement (159 s; *p* = 0.058).

**Table 5:** Rope race completion times relative to sex differences.

Phase	Females *N* = 14		Males *N* = 6		*P* value
	Mean	SD	Mean	SD	
Warm-up	373	161	340	123	*p* = 0.627
Live test 1	200	59	182	85	*p* = 0.639
Live test 2	208	99	158	73	*p* = 0.233
Live test 3*	208	134	140	33	*p* = 0.092

Test 3*.

## 4 Discussion

In this randomised controlled trial, we demonstrated that continuous remote instructor supervision during the initial phase of laparoscopic simulator training significantly reduced perceived difficulty. It also improved task completion times in initial exercises, although not to a significantly greater extent than independent practice. These findings support our primary hypothesis and suggest that real-time guidance serves as a critical catalyst for early skill acquisition in undergraduate medical education. Although supervised participants demonstrated faster early performance and lower perceived difficulty, no statistically significant differences were detected in final performance times. These nonsignificant results must be interpreted cautiously, given the study's limited power and reliance on completion time as the sole objective metric.

Our results align with and extend prior work demonstrating the value of structured simulation in early surgical training (5, 6). Early exploratory studies established that simulator exposure enhances operating room performance (5) and that curriculum-based practice improves novice laparoscopic skills (6). Based on this experience Ahlberg et al. (1) showed that proficiency-based VR training reduced intraoperative errors during early cholecystectomies, underscoring the educational importance of guided, criterion-driven practice. More specifically, the present trial confirms the beneficial impact of real-time supervision: the results reflect the benefits of individualized feedback (3) and remote tele-mentoring (4), both of which underscore the critical role of timely guidance in shaping efficient instrument trajectories and task execution. In contrast to many prior studies that compared training vs. no training, our supervised vs. independent comparison addresses a practical gap in undergraduate curricula, where one-to-one tutoring resources are constrained and must be organized effectively if the supervisor cannot be physically present.

Our findings align with recent advances in simulator design, such as the modular and validated colonoscopy platform described by Finocchiaro et al., which underscores the importance of realistic haptic feedback, structured training progression, and objective performance assessment in simulation-based education (IEEE Access, 2023). Incorporating such principles strengthens the argument that supervision remains an important catalyst during the initial acquisition of technical skills (12).

Recent systematic reviews and meta-analyses reinforce these findings: simulation-based curricula consistently improve operative performance and reduce error rates (9). Moreover, cognitive load theory suggests that structured guidance during early skill acquisition optimizes learning efficiency (10, 11).

Subgroup analyses were consistent with existing literature. Participants with greater gaming experience performed faster in the third exercise, despite no baseline advantage, consistent with findings that visuospatial ability and gaming exposure predict simulator performance and learning curves. Sex-based differences were exploratory and should be interpreted cautiously, given the small sample. Given that the primary outcome was defined as Test 1, the lack of statistically significant between-group differences at this time point must be interpreted in the context of the study's limited power. As noted, the reduced sample size increases the likelihood of Type II error, and nonsignificant findings cannot be interpreted as evidence of equivalence.

Strengths include randomized design, objective time completion metrics, and triangulation with subjective outcomes.

## 5 Limitations

The main limitation of this study was the small sample size (*n* = 20). Due to recruitment challenges, the trial was terminated early. Consequently, the study is underpowered relative to the *a priori* calculation. Although the *a priori* power calculation indicated that 36 participants were required to achieve 70% statistical power, only 20 were ultimately enrolled. *post-hoc* power analysis showed that the achieved power was approximately 28%–35%, substantially increasing the risk of Type II error. Subsequently, we cannot rule out the risk of Type II errors and subtle differences between the groups that may have been missed. However, the consistent direction of the effect sizes—favouring supervision across subjective and objective measures—suggests a genuine pattern rather than a random effect. Furthermore, the single-centre design and the absence of delayed retention tests limit the generalisability of findings regarding long-term skill maintenance. An additional limitation is the loss of detailed kinematic performance metrics due to a storage malfunction in the simulator software. As a result, only task completion time could be analyzed.

## 6 Conclusion and educational implications

Despite the sample size constraints, this study provides preliminary evidence that remote real-time supervision is superior to unsupervised practice for introducing novices to laparoscopic surgery. The data supports a hybrid curricular model: resource-intensive supervision (or tele-mentoring) should be front-loaded during the “warm-up” and initial procedural attempts to establish correct psychomotor schemas and reduce frustration. Although, Given the absence of statistically significant differences in final task performance and the study's limited sample size, our findings support supervised training primarily as a beneficial early-phase intervention rather than conclusive evidence of superior overall performance outcomes. Once basic competence is achieved, learners may move on to independent, metric-based volume training. As demonstrated by Oussi et al. (4), tele-mentoring offers a scalable solution to the resource bottlenecks traditionally associated with supervised training. Future adequately powered trials should focus on the cost-effectiveness of such hybrid models and their impact on skills transfer to the operating theatre.

## Data Availability

The raw data supporting the conclusions of this article will be made available by the authors, without undue reservation.
